# Supplementary material for: Content-rich biological network constructed by mining PubMed abstracts
Source: BMC Bioinformatics. 2004 Oct 8;5:147. doi: 10.1186/1471-2105-5-147 (PMC528731; doi:10.1186/1471-2105-5-147)
Supplement: Additional File 2 — The original results of the above study (non-essential files are deleted to keep the file size under the limit set by BMC bioinformatics). [file 1471-2105-5-147-S2.bz2 › chilibotAdditionalFile2/dip40/352ID7834747E4685/html/BCL2_BAG1.html]

 


 **BCL2** and **BAG1** 
  
Found 100 abstracts in PubMed, retrieved 40.  
 

 What does Google say? 
 PDF only 
| .edu only 

---

**Interactive relationship** (e.g. stimulation, inhibition, etc)

**Neutral relationship**- The expression of antiapoptotic Bcl 2  [ **BCL2** ]  Bag 1  [ **BAG1** ]  proteins was up regulated in the CoPP group.  Ref: 11821745 Transplantation, 2002
- BAG 1  [ **BAG1** ]  is a recently identified Bcl 2  [ **BCL2** ]  interacting anti apoptotic protein.  Ref: 12402153 Br J Cancer, 2002
- Bag 1  [ **BAG1** ]  is a recently identified bcl 2  [ **BCL2** ]  related protein and is known to be linked to the biological aggressiveness of some carcinomas.  Ref: 12680148 Anticancer Res< MedlineTA>Anticancer Res, 2002
- For example, Bcl 2  [ **BCL2** ] , Bag 1  [ **BAG1** ]  and Bak levels increased in the colon, but the levels of these same homologs decreased in the ileum.  Ref: 11332706 Histol Histopathol, 2001
- Bcl 2  [ **BCL2** ]  associated athanogene BAG  [ **BAG1** ]  family proteins are BAG  [ **BAG1** ]  domain containing proteins that interact with the heat shock proteins 70, both constitutive Hsc70 and inducible Hsp70.  Ref: 12406544 Cancer Lett, 2002
- In addition, several proteins that are not members of the Bcl 2  [ **BCL2** ]  family can bind to Bcl 2  [ **BCL2** ] , including BAG 1  [ **BAG1** ]  protein.  Ref: 11527400 Biochem Biophys Res Commun, 2001
- Bag 1  [ **BAG1** ]  exerts powerful antiapoptotic effects by binding and stabilizing Bcl 2  [ **BCL2** ]  and interacting with the tumor necrosis factor receptor type I induced death signal.  Ref: 12215270 Hum Gene Ther, 2002
- In BAG  [ **BAG1** ]  3 and BAG  [ **BAG1** ]  4 SODD, the BAG  [ **BAG1** ]  domains appear to constitute the Bcl 2  [ **BCL2** ]  binding regions of these molecules.  Ref: 11527400 Biochem Biophys Res Commun, 2001
- Here, we found that honokiol induced apoptotic cell death was accompanied by upregulation of Bad and downregulation of Bcl XL, while honokiol had no effect on the levels of Bcl 2  [ **BCL2** ] , Bcl XS, Bag 1  [ **BAG1** ] , Bax and Bak proteins.  Ref: 12007567 Biochem Pharmacol, 2002
- It was revealed that LIGHT treatment resulted in down regulation of anti apoptosis Bcl 2  [ **BCL2** ]  family member Bcl 2  [ **BCL2** ] , Bcl X L, Bag 1  [ **BAG1** ] , and Mcl 1.  Ref: 12767529 Cancer Lett, 2003
- Of particular relevance to tumour cells, BAG 1  [ **BAG1** ]  interacts with the anti apoptotic BCL 2  [ **BCL2** ]  protein, various nuclear hormone receptors and the 70 kDa heat shock proteins, Hsc70 and Hsp70.  Ref: 12618309 Biochim Biophys Acta, 2003
- The predictive value of bcl 2  [ **BCL2** ] , bax, bcl xL, bag 1  [ **BAG1** ] , fas, and fasL for chemotherapy response in advanced breast cancer.  Ref: 11895913 Clin Cancer Res, 2002
- BAG 1  [ **BAG1** ]  was originally identified as a binding partner of anti apoptotic factor Bcl 2  [ **BCL2** ]  Takayama et al.  Ref: 12565851 Biochem Biophys Res Commun, 2003
- gossypol induced apoptosis triggers down regulation of anti apoptosis Bcl 2  [ **BCL2** ]  members Bcl X L, Bag 1  [ **BAG1** ]  and Mcl 1, up regulation of pro apoptosis Bcl 2  [ **BCL2** ]  member Bak, activation of caspase,, and 9, up regulation of Apaf 1, release of cytochrome c cyto c from mitochondria, and activation of both DFF45 and PARP.  Ref: 12818369 Biochem Pharmacol, 2003
- Both BAG  [ **BAG1** ]  3 and BAG  [ **BAG1** ]  4 SODD were found to physically associate with Bcl 2  [ **BCL2** ] , and both proteins are well conserved from human to mouse.  Ref: 11527400 Biochem Biophys Res Commun, 2001
- Aloe emodin induced apoptosis of CH27 cells involved modulation of the expression of Bcl 2  [ **BCL2** ]  family proteins, such as BclX L, Bag 1  [ **BAG1** ] , and Bak, and was associated with the translocation of Bak and Bax from cytosolic to particulate fractions.  Ref: 11730720 Eur J Pharmacol, 2001
- Both BAG 1  [ **BAG1** ]  and BAG  [ **BAG1** ]  3 CAIR 1 interact with Bcl 2  [ **BCL2** ]  and have been shown to have a supra additive anti apoptotic effect with Bcl 2  [ **BCL2** ] .  Ref: 12406544 Cancer Lett, 2002
- Together, our findings demonstrate that other members of the BAG 1  [ **BAG1** ]  protein family, namely BAG  [ **BAG1** ]  3 and BAG  [ **BAG1** ]  4 SODD, bind to Bcl 2  [ **BCL2** ]  and provide a potential link between pathways regulated by Bcl 2  [ **BCL2** ]  and pathways regulated by Hsp70, as well as TNFR1.  Ref: 11527400 Biochem Biophys Res Commun, 2001
- The anti apoptotic activities of BAG  [ **BAG1** ]  family proteins may be dependent on their interactions with Hsc70 Hsp70 and or binding to Bcl 2  [ **BCL2** ] .  Ref: 12406544 Cancer Lett, 2002
- BAG  [ **BAG1** ]  3 Bis, CAIR, which was identified as a BAG 1  [ **BAG1** ]  related protein, is a novel modulator of cellular anti apoptotic activity that functions through its interaction with Bcl 2  [ **BCL2** ] .  Ref: 11513873 FEBS Lett, 2001
- BAG 1  [ **BAG1** ]  BCL 2  [ **BCL2** ]  athanogene 1, a multifunctional protein which associates with steroid hormone receptors including the oestrogen receptor and the anti apoptotic BCL 2  [ **BCL2** ]  protein, regulates steroid hormone dependent transcription and apoptosis.  Ref: 12081204 J Pathol, 2002
- CONCLUSION These findings suggest that bag 1  [ **BAG1** ]  may interact with bcl 2  [ **BCL2** ]  to play an important role in thyroid neoplasm before anaplastic transformation, and the disruption of events mediated by bag 1  [ **BAG1** ]  and bcl 2  [ **BCL2** ]  may be a typical characteristic of undifferentiated carcinoma.  Ref: 12680148 Anticancer Res< MedlineTA>Anticancer Res, 2002
- Isolation of Bcl 2  [ **BCL2** ]  binding proteins that exhibit homology with BAG 1  [ **BAG1** ]  and suppressor of death domains protein.  Ref: 11527400 Biochem Biophys Res Commun, 2001
- The clonogenic growth of breast cancer cell lines was inhibited by a ligand for PPARgamma troglitazone, TGZ combined with a ligand for either retinoid X receptor RXR LG10069 4 8 cell lines, RAR ATRA 5 8 cell lines or RAR RXR and RXR RXR 9 cis RA 5 8 cell lines independent of their expression of bcl 2  [ **BCL2** ] , bag 1  [ **BAG1** ] , ERalpha, and p53.  Ref: 12186376 Breast Cancer Res Treat, 2002
- In 126 patients, histological blocks of primary tumors were available for immunohistochemical analysis of bax, bcl 2  [ **BCL2** ] , bcl xL, bag 1  [ **BAG1** ] , fas and fasL.  Ref: 11895913 Clin Cancer Res, 2002
- While Bcl 2  [ **BCL2** ]  levels were permanently and Bag 1  [ **BAG1** ]  levels transiently elevated upon therapy, other apoptosis regulatory genes revealed no alterations.  Ref: 12667663 J Neuroimmunol, 2003
- Bis also called Bag  [ **BAG1** ]  3, identified as a novel Bcl 2  [ **BCL2** ]  interacting protein, has been shown to enhance anti cell death activity of Bcl 2  [ **BCL2** ] .  Ref: 12061864 Exp Neurol, 2002
- BAG 1  [ **BAG1** ]  also known as RAP46 HAP46 was originally identified as a 46 kDa protein that bound to and enhanced the anti apoptotic properties of Bcl 2  [ **BCL2** ] .  Ref: 11494137 Oncogene, 2001

**Non-interactive relationship** (e.g. studied together, co-existance, homology, etc.)

- Bag 1  [ **BAG1** ]  expression in thyroid neoplasm its correlation with Bcl 2  [ **BCL2** ]  expression and carcinoma dedifferentiation.  Ref: 12680148 Anticancer Res< MedlineTA>Anticancer Res, 2001
- The present study was performed to clarify the clinical implications of BAG 1  [ **BAG1** ] , a bcl 2  [ **BCL2** ]  binding protein in esophageal squamous cell carcinoma ESCC.  Ref: 12823208 Dis Esophagus, 2003
- These in vitro results suggest that distinct isoforms of BAG 1  [ **BAG1** ]  have different anti apoptotic functions and their functions may be correlated to increased Bcl 2  [ **BCL2** ]  expression.  Ref: 12370827 Oncogene, 2002
- Intragraft expression of antiapoptotic Bcl 2  [ **BCL2** ]  and Bag 1  [ **BAG1** ]  was increased in Ad HO 1 treated orthotopic liver transplants, as compared with Ad beta Gal controls.  Ref: 12134106 Transplantation, 2002
- BAG  [ **BAG1** ]  Bcl 2  [ **BCL2** ]  associated athanogene proteins are molecular chaperone regulators that affect diverse cellular pathways.  Ref: 12058034 J Biol Chem, 2002
- Four anti apoptotic proteins were altered 2.1 to 14.6 fold for Bcl 2  [ **BCL2** ]  and BAG 1  [ **BAG1** ]  isoform p33 and p29.  Ref: 12706450 Eur J Pharmacol, 2003
- For instance, Bag 1  [ **BAG1** ]  and Bcl 2  [ **BCL2** ]  exhibited crypt villus decreasing gradients of expression in the ileum but not in the colon.  Ref: 11332706 Histol Histopathol, 2001
- In contrast, phosphorylation of Bcl 2  [ **BCL2** ]  induced by microtubule targeting drugs did not alter its ability to associate with Bcl 2  [ **BCL2** ]  homodimerization, Bax,  **BAG1** , or other Bcl 2  [ **BCL2** ]  binding proteins.  Ref: 11774038 Neoplasia< MedlineTA>Neoplasia, 2001
- In the present study, we approached this by investigating the expression of six Bcl 2  [ **BCL2** ]  homologs Bcl 2  [ **BCL2** ] , Bcl XL, Mcl 1, Bax, Bak, Bad, and one nonhomologous associated molecule Bag 1  [ **BAG1** ] , during development of the human ileum and colon 12 20 wks of gestation.  Ref: 11332706 Histol Histopathol, 2001
- The hsp70 hsc70 associating protein Hap46 of human origin, also called BAG  [ **BAG1** ]  1M Bcl 2  [ **BCL2** ]  associated athanogene 1, has been characterized previously as a DNA binding protein, which is able to stimulate transcription.  Ref: 12682371 Nucleic Acids Res, 2003
- BAG  [ **BAG1** ]  1M, an isoform of Bcl 2  [ **BCL2** ]  interacting protein BAG 1  [ **BAG1** ] , enhances gene expression driven by CMV promoter.  Ref: 11520069 Biochem Biophys Res Commun, 2001
- This screen revealed the elevated expression of BCL 2  [ **BCL2** ] , BCL 2A1, BAG 1  [ **BAG1** ]  and TACE upon stimulation with GM CSF.  Ref: 12648514 Leuk Res, 2003
- Peripheral immune cells were investigated for apoptosis and for the expression of apoptosis regulatory genes CD95, CD95 ligand, FLIP, Bcl 2  [ **BCL2** ] , Bcl X L, Bag 1  [ **BAG1** ] , and caspase 3 by quantitative real time PCR.  Ref: 12667663 J Neuroimmunol, 2003
- RESULTS Of the investigated factors, bag 1  [ **BAG1** ]  correlated positively with bax, bcl 2  [ **BCL2** ] , and fasL, and fasL correlated positively with fas and bax.  Ref: 11895913 Clin Cancer Res, 2002
- To determine whether changes in the expression of proteinases, 73 kDa constitutive heat shock cognate protein Hsc70 and stress inducible 72 kDa heat shock protein Hsp70 Hsc Hsp70, and Bcl 2  [ **BCL2** ]  associated gene product 1 BAG 1  [ **BAG1** ]  contribute to the remodeling response of muscle tissue, tibialis anterior muscles of male Sprague Dawley rats were injected with 0.75% bupivacaine and removed at, 1, or 35 days postinjection n = 5 7 group.  Ref: 12791605 Am J Physiol Endocrinol Metab, 2003
- In this study, we screened for proteins that bind to Bcl 2  [ **BCL2** ] , and isolated two additional members of the BAG 1  [ **BAG1** ]  protein family, BAG  [ **BAG1** ]  3 and BAG  [ **BAG1** ]  4.  Ref: 11527400 Biochem Biophys Res Commun, 2001
- ... other Bcl 2  [ **BCL2** ]  family proteins including Bax, Bcl 2  [ **BCL2** ] , Bcl XL, and Bag  [ **BAG1** ]  remained unchanged.  Ref: 12874837 J Cell Biochem, 2003
- Results from coimmunoprecipitation and colocalization studies indicate that this molecular chaperone forms complexes with other molecular chaperones and cochaperones, including Hsp90, Hsp40, the Hsp70 Hsp90 organizing protein Hop, the Hsp70 interacting protein Hip, and the  **Bcl2**  associated athanogene 1  [ **BAG1** ]  protein BAG 1  [ **BAG1** ] .  Ref: 11559757 J Cell Sci, 2001
- MATERIALS AND METHODS We immunohistochemically investigated bag 1  [ **BAG1** ]  and bcl 2  [ **BCL2** ]  expression in various thyroid neoplasms using monoclonal antibodies.  Ref: 12680148 Anticancer Res< MedlineTA>Anticancer Res, 2001
- These data implicate induction of the stress response and its modulation by the chaperone cofactor and Bcl 2  [ **BCL2** ]  family member BAG 1  [ **BAG1** ] , after TBI in humans.  Ref: 12558971 J Neurochem, 2003
- Down regulation of Bcl 2  [ **BCL2** ]  interacting protein BAG 1  [ **BAG1** ]  confers resistance to anti cancer drugs.  Ref: 12565851 Biochem Biophys Res Commun, 2003
- The expression of Bis also called Bag  [ **BAG1** ]  3, a Bcl 2  [ **BCL2** ]  binding protein, was investigated in the rat kainic acid KA model of temporal lobe epilepsy.  Ref: 12085992 Exp Mol Med, 2002
- Our data demonstrated that Gyp induced apoptotic cell death was accompanied by up regulation of Bax, Bak and Bcl X, and down regulation of Bcl 2  [ **BCL2** ]  and Bad, while it had no effect on the level of Bag 1  [ **BAG1** ]  protein.  Ref: 12065092 Cancer Lett, 2002
